# A Combination of Two Probiotics, *Lactobacillus sporogenes* and *Clostridium butyricum*, Inhibits Colon Cancer Development: An In Vitro Study

**DOI:** 10.3390/microorganisms10091692

**Published:** 2022-08-23

**Authors:** Oana Budu, Christian Banciu, Iulia Pinzaru, Cristian Sarău, Daniel Lighezan, Codruța Șoica, Cristina Dehelean, George Drăghici, Alina Dolghi, Alexandra Prodea, Marius Mioc

**Affiliations:** 1 Department of Internal Medicine IV, Faculty of Medicine, “Victor Babes” University of Medicine and Pharmacy Timisoara, Eftimie Murgu Square No. 2, 300041 Timişoara, Romania; 2 Department of Toxicology and Drug Industry, Faculty of Pharmacy, “Victor Babes” University of Medicine and Pharmacy Timisoara, Eftimie Murgu Square No. 2, 300041 Timişoara, Romania; 3 Research Centre for Pharmaco-Toxicological Evaluation, “Victor Babes” University of Medicine and Pharmacy, Eftimie Murgu Sq. No. 2, 300041 Timisoara, Romania; 4 Department of Medical Semiology I, Faculty of Medicine, “Victor Babes” University of Medicine and Pharmacy Timisoara, Eftimie Murgu Square No. 2, 300041 Timişoara, Romania; 5 Department of Pharmaceutical Chemistry, Faculty of Pharmacy, “Victor Babes” University of Medicine and Pharmacy Timisoara, Eftimie Murgu Square No. 2, 300041 Timişoara, Romania

**Keywords:** probiotics, 5-fluorouracil, *Lactobacillus sporogenes*, *Clostridium butyricum*, colon cancer, lung cancer, liver cancer, apoptosis

## Abstract

Cancer remains a leading cause of death worldwide and, even though several advances have been made in terms of specific treatment, the late-stage detection and the associated side effects of the conventional drugs sustain the search for better treatment alternatives. Probiotics are live microorganisms that have been proven to possess numerous health benefits for human hosts, including anticancer effects. In the present study, the in vitro effect of the association of two probiotic strains (PBT), *Lactobacillus sporogenes* and *Clostridium butyricum*, were tested against colon (HT-29 and HCT 116), lung (A549), and liver (HepG2) cancer cell lines, alone or in combination with 5-fluorouracil (5FU). Moreover, the underlying mechanism of PBT and PBT-5FU against the HT-29 cell line was evaluated using the Hoechst 33342 staining, revealing characteristic apoptotic modifications, such as chromatin condensation, nuclear fragmentation, and membrane blebbing. Furthermore, the increase in the expression of pro-apoptotic Bax, Bid, Bad, and Bak proteins and the inhibition of the anti-apoptotic Bcl-2 and Bcl-XL proteins were recorded. Collectively, these findings suggest that the two strains of probiotic bacteria, alone or in association with 5FU, induce apoptosis in colon cancer cells and may serve as a potential anticancer treatment.

## 1. Introduction

Cancer is a leading cause of death globally; the year 2020 marked nearly 10 million fatal cases, with the most common being lung (1.8 million), colon and rectum (916,000), and liver (830,000) cancer-related deaths. In 2017, the World Health Assembly passed a resolution (WHA70.12) urging governments to take immediate action in order to reach the objectives of the 2030 UN Agenda for Sustainable Development, which aims at the reduction in cancer-related deaths [1]. The last decades saw many cancers cured if detected in the early stages and if effective treatment was applied. However, effective treatment continues to be a matter of debate despite the huge progress of anticancer therapies, depending on the cancer type, location, and stage, as well as on the patient’s health status. The most effective therapy is surgery in the early stages of tumor development; unfortunately, when cancer is detected in the late stages, systemic therapy is needed either as a single treatment or as a preliminary step before surgery. Systemic therapy, whether it is chemotherapy or immunotherapy, comes with a plethora of side effects or other inconveniences; therefore, patients often search for alternatives and more natural treatments to act either as a curative or a preventive against cancer development or relapse.

Probiotics are live microorganisms that in proper amounts may provide health benefits for the host; they have been proven as effective in improving the immune system and intestinal health [2]. In addition, probiotics exhibit anticancer, antidiabetic, antioxidant, and antibacterial activities [3]. Probiotics have also been introduced as functional food ingredients, with *Lactobacillus* and *Bifidobacterium* being the most commonly used genera, followed by *Bacteroides* and *Clostridium* [4].

Probiotics have revealed their therapeutic benefits in the chemoprevention of cancer or as adjuvants during cancer chemotherapy; most in vitro studies have been conducted on gastric and colon cancer cells where various probiotics decreased cell proliferation and induced apoptosis. However, similar effects were reported on some systemic cancer cells, mainly leukemia and lymphoma cells; additionally, several clinical studies emphasized probiotics’ efficacy in stopping cancer progression in various cancer patients (colorectal, liver, breast, bladder, and uterus) [5]. Further in-depth studies identified several specific molecular mechanisms induced by probiotics, such as oncogene downregulation, kinase inhibition, and tumor suppressor reactivation [6]. Despite their multitude of benefits, probiotics display several limitations as well, such as insufficiently identified molecular mechanisms, the triggering of antibiotic resistance, competition with the commensal gut microflora, the potential for opportunistic infections, and paradoxical inflammatory effects. Therefore, further studies are recommended, with both in vitro and clinical trials, in order to identify specific strains with benefits against particular cases of cancer; moreover, taking into consideration that the majority of current studies focus on gastrointestinal malignancies, more studies should be conducted on the molecular mechanisms of probiotics in other types of cancer.

5-fluorouracil (5FU) is a chemotherapeutic frequently employed in the treatment of different types of cancer, such as colon, breast, stomach, esophageal, skin, and pancreatic cancers [7]. 5FU is an analogue of uracil that is able to penetrate the cell membrane through the same transport mechanism as uracil, and it is further transformed in several active metabolites that inhibit the thymidylate synthase and disrupt the synthesis of RNA, leading to the apoptosis of cancer cells [8]. The emergence of resistance in tumors exposed to 5FU currently limits its use as a stand-alone therapy [9] and determines the search for other therapeutic alternatives, including those associated with probiotic strains [10].

In the current study, two probiotic strains, *Lactobacillus sporogenes* and *Clostridium butyricum*, were tested as anticancer agents, alone or in combination with 5FU, against colon, lung, and liver cancer cells, respectively; their efficacy is assessed in a comparative manner in order to identify the potentially different mechanisms against gastrointestinal versus lung and liver cancer.

## 2. Materials and Methods

### 2.1. Reagents, Bacteria, and Cell Lines

Phosphate saline buffer (PBS), fetal bovine serum (FBS), penicillin/streptomycin mixture, trypsin-EDTA solution, dimethyl sulfoxide (DMSO), 3-(4,5-dimethylthiazol-2-yl)-2,5-diphenyltetrazolium bromide (MTT), *Lactobacillus sporogenes*, and *Clostridium butyricum* TO-A were purchased from American Type Cell Collection (Lomianki, Poland), Sigma Aldrich, Merck KgaA (Darmstadt, Germany). The cell culture media, McCoy’s 5A Medium (ATCC^®^ 30-2007™), Eagle’s Minimum Essential Medium (EMEM-ATCC^®^ 30-2003™), and DMEM were purchased from ATCC (American Type Cell Collection, Lomianki, Poland). All the reagents corresponded to the analytical standard purity and were applied according to the manufacturers’ recommendations.

The bacterial strains were cultured under proper anaerobic conditions in MRS and BHI broth, respectively, following the steps described in the literature: (i) incubation at 37 °C for 24 h; (ii) centrifugation at 3500 rpm for 10 min; (iii) washing with PBS; and (iv) resuspension in the PBS and adjustment of the optical density (OD_600_) to correspond to 10^7^ CFU/mL (colony-forming units per milliliter) (LogPhase 600, Microbiology Reader, BioTek Instruments Inc., Winooski, VT, USA) [11,12].

Four tumoral cell lines were selected for the current study, namely colorectal adenocarcinoma (HT-29, ATCC^®^ HTB-38TM), colorectal carcinoma (HCT 116, ATCC^®^ CCL-247TM), human hepatocellular carcinoma (HepG2, ATCC^®^ HB-8065™), and human lung carcinoma (A549, ATCC^®^ CCL-185™), which were purchased from ATCC (American Type Cell Collection) as frozen vials. The cell culture involved the following steps: (i) specific media addition—for HT-29 and HCT 116 cells McCoy’s 5A Medium (ATCC^®^ 30-2007™), for HepG2 Eagle’s Minimum Essential Medium (EMEM—ATCC^®^ 30-2003™), and for A549 DMEM; (ii) supplementation with 10% FBS and 1% antibiotic mixture (100 U/mL penicillin/100 µg/mL streptomycin); and (iii) standard conditions—incubation in a humidified atmosphere at 37 °C and 5% CO_2_.

### 2.2. Cell Viability

The cell viability was assessed using the MTT (3-(4,5-dimethylthiazol-2-yl)-2,5-diphenyltetrazolium bromide) assay, as presented in our previous study [13]. Briefly, the cells were cultured in 96-well plates (10,000 cells/200 µL/well) and treated with test samples (5FU, PBT, and PBT-5FU), followed by the incubation period of 24, 48, and 72 h, respectively. In the ensuing treatment period with the different samples (5FU, PBT, and PBT-5FU), 10 µL/well of MTT solution (5 mg/mL) was added in each well, and the plate was incubated for 3 h, whereupon the formed formazan crystals were dissolved in 100 µL of solubilization buffer provided by the manufacturer for 30 min, in the dark. Finally, the reduced MTT was spectrophotometrically analyzed at 570 and 630 nm, using the Cytation 5 (BioTek Instruments Inc., Winooski, VT, USA) microplate reader. All experiments were performed in triplicate.

### 2.3. Cells’ Morphology Assessment

To determine the cytotoxic potential of the test samples, a microscopic evaluation of the cells’ morphology and shape was performed. The cells (10,000 cells/200 µL/well) were observed under bright field illumination and photographed at 24 h after treatment and compared with the solvent (media). The photos were taken using Cytation 1 (BioTek Instruments Inc., Winooski, VT, USA). The analysis of the images was performed by means of the Gen5™ microplate data collection and analysis software (BioTek Instruments Inc., Winooski, VT, USA).

### 2.4. Nuclear Morphology

The potential toxicity of the samples (5FU, PBT, and PBT-5FU) at the nuclear level was evaluated by using the Hoechst 33342 staining assay protocol according to the manufacturer’s (Thermo Fisher Scientific, Inc., Waltham, MA, USA) recommendations and to our previous research [14]. In brief, the malignant cells were seeded in 12-well plates (100,000 cells/1.5 mL/well) and treated with test samples in solvent (media) for 24 h. After the stimulation period, the media was removed, and the staining solution diluted at 1:2000 in PBS was added (500 µL/well). The plates were incubated for 10 min at room temperature, protected from light. Finally, the staining solution was washed with PBS, and the pictures were taken using Cytation 1 (BioTek Instruments Inc., Winooski, VT, USA) and analyzed by the means of the Gen5™ Microplate Data Collection and Analysis Software (BioTek Instruments Inc., Winooski, VT, USA). Staurosporine (STP) 5 µM was selected as a positive control for apoptosis.

### 2.5. Gene Expression

Given that, following the cell viability test, the most affected cell line was HT-29, it was decided that the influence of 5FU, PBT, and PBT-5FU on gene expression should be established by applying the RT-PCR method [15] to this cell line. To evaluate the expression of the Bax, Bcl-2 (Thermo Fisher Scientific, Inc., Waltham, MA, USA), and Bad (Eurogentec, Seraing, Belgium), the cells (1,000,000 cells/well) were cultured in 6-well plates. After reaching a confluence of approximately 80%, the cells were stimulated with test samples for a period of 72 h. After this time, RNA was isolated using Trizol reagent and the Quick-RNA™ purification kit, and its amount was determined using a DS-11 spectrophotometer (DeNovix, Wilmington, DE, USA). Finally, RNA transcription was completed using the Maxima^®^ First Strand cDNA Synthesis Kit, and quantitative real-time PCR analysis was performed using the Quant Studio 5 real-time PCR system (Thermo Fisher Scientific, Inc., Waltham, MA, USA) in the presence of Power SYBR-Green PCR Master Mix.

### 2.6. Statistical Analysis

The data were processed as means ± standard deviation (SD). The software GraphPad Prism version 6.0.0 for Windows (GraphPad Software, San Diego, CA, USA, www.graphpad.com, accessed on 13 July 2022) was used. The differences between the data were compared by performing the one-way ANOVA analysis and Dunett’s multiple comparisons post-test. The statistically significant differences between the data were labeled with * (* *p* < 0.05; ** *p* < 0.01; *** *p* < 0.001; **** *p* < 0.0001).

## 3. Results

### 3.1. Cell Viability Evaluation

**Figure 1:**
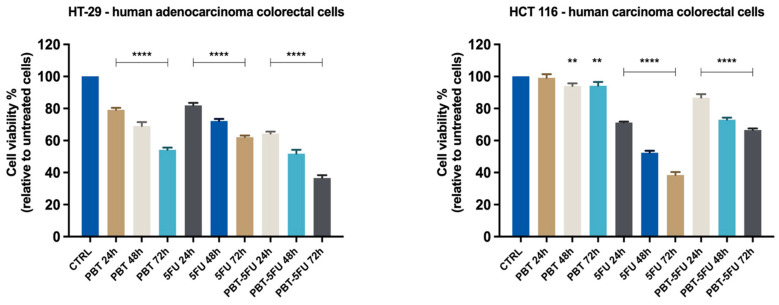
In vitro evaluation of the effect exerted by PBT (*Lactobacillus sporogenes*:*Clostridium butyricum* TO-A = 25:1, 10^7^ million CFU), 5FU (5-fluorouracil 25 μM), and PBT-5FU after 24, 48, and 72 h of treatment on HT-29 and HCT 116 cells’ viability by performing the MTT assay. Data are presented as viability percentages (%) normalized to a control (untreated cells) and expressed as mean values ± SD of three independent experiments performed in triplicate. The statistical differences between untreated and the treated cells were analyzed by applying the one-way ANOVA method followed by Dunnett’s multiple comparisons post-test (** *p* < 0.01; **** *p* < 0.0001).

In order to analyze the capacities of PBT, 5FU, and their associations to inhibit cell proliferation, the 3-4,5-dimethylthiazol-2-yl-2,5-diphenyltetrazolium bromide (MTT) assay was performed. Three samples (PBT-10^7^ CFU, 5FU-25 μM, and PBT-5FU) were tested on the HT-29, HCT 116, HepG2, and A549 cell lines for 24, 48, and 72 h. In all cases, the viability percentages varied in a sample type manner, PBT displaying an actual anti-cancer effect only in adenocarcinoma colorectal cells. In HT29 cells, the cytotoxic activity of PBT alone increased in a time-dependent manner (Figure 1), with the cell viability varying from 57% to 80% after 72 and 24 h, respectively. In these cells, the cytotoxic effect of 5FU was significantly lower compared to PBT, ranging from 68% to 81% after 72 and 24h, respectively, following a similar time-dependent manner. When the two agents were combined, the overall cytotoxicity was clearly improved; after 24h, cell viability was 70%; 55% after 48 h; and 38% after 72h. The coefficient of drug interaction (CDI) was used to analyze the interactions between the individual agents while used as a mixture. The coefficient of drug interaction (CDI) was calculated by using the formula:

CDI = AB/(A × B),

where AB represents the ratio between the absorbancy values of the mixture (PBT + 5FU) and the control groups, while A or B is the ratio between the absorbancy values of the single agent and the control group; the results are shown in Table 1. According to the CDI values, the interactions were categorized as synergism, additivity, or antagonism, respectively, as follows: a CDI value of <1, =1, or >1 indicates that the agents are synergistic, additive, or antagonistic, respectively [16]. One can notice that in HT29 cells, for all three time intervals, the CDI values reach around 1, thus indicating an additive interaction between the two tested agents.

**Figure 2:**
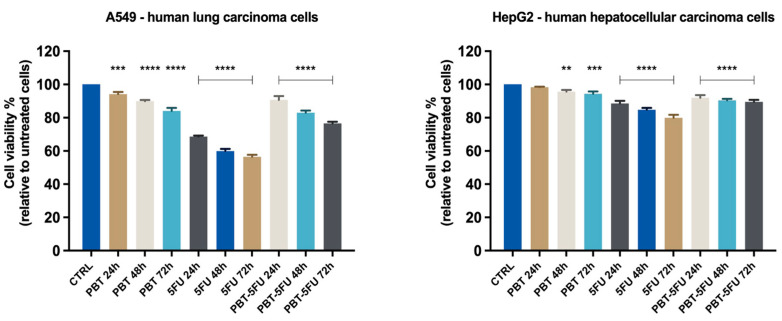
In vitro evaluation of the effect exerted by PBT (*Lactobacillus sporogenes*:*Clostridium butyricum* TO-A = 25:1, 10^7^ million CFU), 5FU (5-fluorouracil 25 μM), and PBT-5FU after 24, 48, and 72 h of treatment on A549 and HepG2 cells’ viability by performing the MTT assay. Data are presented as viability percentages (%) normalized to a control (untreated cells) and expressed as mean values ± SD of three independent experiments performed in triplicate. The statistical differences between untreated and the treated cells were analyzed by applying the one-way ANOVA method followed by Dunnett’s multiple comparisons post-test (** *p* < 0.01; *** *p* < 0.001 **** and *p* < 0.0001).

In the HCT116 cells, PBT completely lacks cytotoxic properties, with the cell viability reaching similar values to the control sample (Figure 1). In turn, 5FU is highly cytotoxic in a time-dependent manner, and after 72h, the cell viability was reduced to less than 40%. The application of the PBT-5FU combination led to unfavorable effects, with the PBT apparently antagonizing the cytotoxic activity of the 5FU. Indeed, the CDI values calculated for all three time intervals are >1, thus indicating an antagonistic combination. Similar results and antagonistic effects were recorded for the lung carcinoma cells. In the HepG2 liver cancer cells, neither agent showed cytotoxic effects; even the small cytotoxic effect of 5FU was counteracted by PBT, with the cell viability recorded for the PBT-5FU combination being in fact similar to the one reported for the control (Figure 2).

### 3.2. Cell Morphology and Confluence

**Figure 3:**
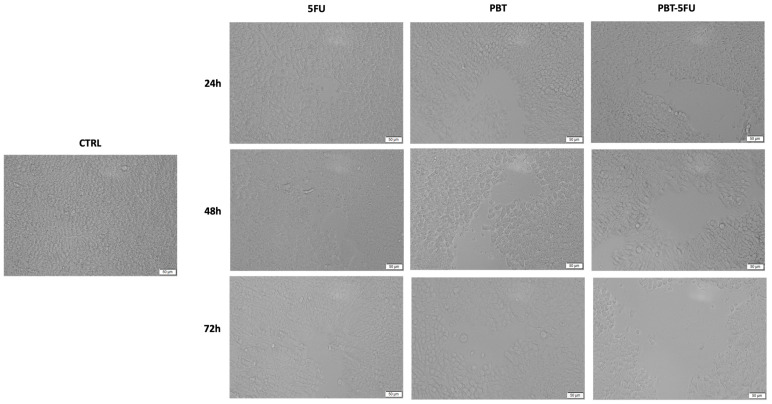
Morphological and shape changes produced by 5FU, PBT and PBT-5FU in HT-29 cells after 24, 48, and 72 h of treatment. The scale bars indicate 50 µm.

As a component of the anti-cancer profile of PBT, a microscopic examination of the HT-29 malignant cells (Figure 3) was performed at the end of the 24, 48, and 72 h of treatment.

Cell morphology changes were recorded after treatment with 5FU at all three time intervals, including the rounding of cells and a decrease in confluency, with the most significant changes occurring after 72 h. Similarly, PBT induced significant cell morphological alterations at all three time intervals, but especially after 72 h. Interestingly, PBT-5FU showed the most pronounced cytotoxicity, in which morphological changes characteristic of cell death were observed at 72 h, accompanied by a decrease in cell confluence. These modifications in the cells’ morphology express clear signs of cytotoxicity and confirm the cells’ viability assessment results (Figure 3).

### 3.3. Nuclear Morphology Assessment

**Figure 4:**
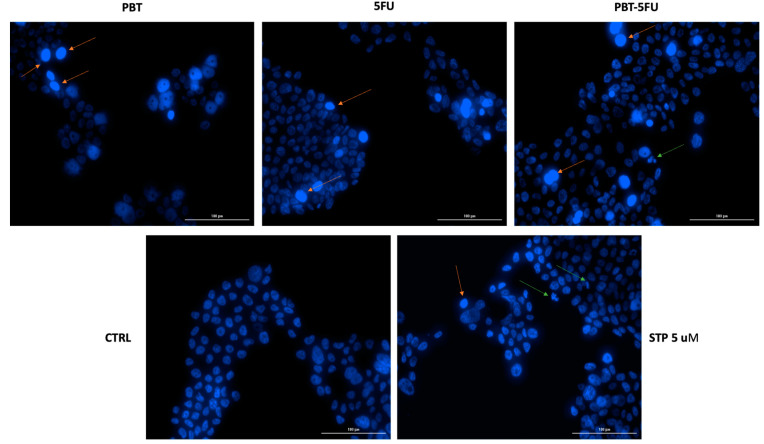
Hoechst 33342 staining of HT-29 cells’ nuclei following the 24 h treatment with: PBT, 5FU, and PBT-5FU. The orange and green arrows indicate nuclei expressing abnormal features. The scale bars represent 100 µm.

As specific changes in the morphology of cell nuclei offer insights into the possible cell death mechanisms induced by anticancer compounds, a Hoechst 33342 staining was conducted for PBT, 5FU, and PBT-5FU. Staurosporine (STP) 5 µM was selected as an indicator for apoptosis. Several apoptotic features were noticed. In HT-29 cells, PBT and 5FU induced chromatin condensation, while PBT-5FU produced chromatin condensation, nuclear fragmentation, and membrane blebbing (Figure 4).

### 3.4. Expression of Apoptotic Markers

**Figure 5:**
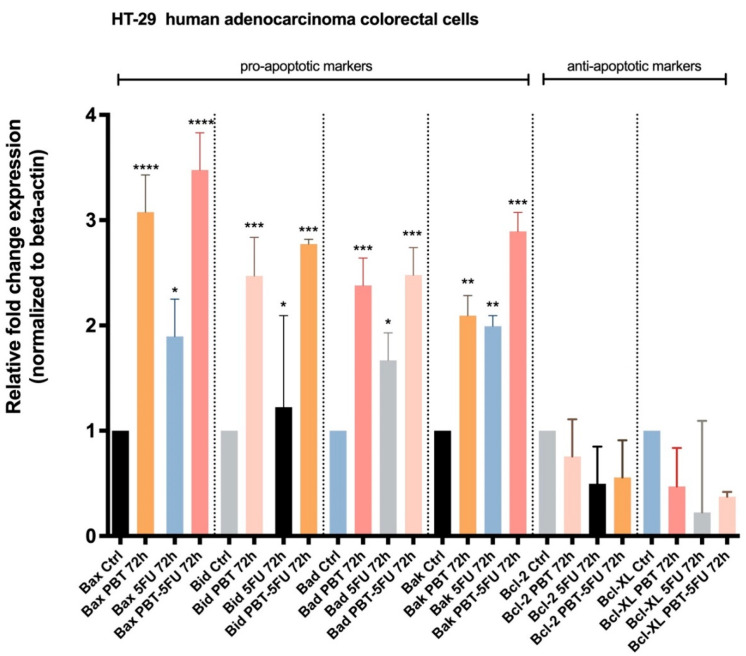
Relative fold change expression of mRNA of pro-apoptotic (Bax, Bid, Bad, and Bak) and anti-apoptotic (Bcl-2 and Bcl-XL) markers in human adenocarcinoma colorectal cells (HT-29)—72 h after exposure to PBT, 5FU, and PBT-5FU. mRNA expression levels normalized to beta-actin expression; mean values ± SD of three independent experiments presented; one-way ANOVA with Tukey’s post-test used to identify the statistical differences (* *p* < 0.05, ** *p* < 0.01, *** *p* < 0.001, and **** *p* < 0.0001).

With regard to the data obtained in the viability cell assessments on human adenocarcinoma colorectal cells, HT-29 highlighted an important decrease in cell viability after the sample (PBT, 5FU, and PBT-5FU) treatment. To obtain more detailed information regarding the mode of action of PBT, 5FU, and PBT-5FU on colorectal adenocarcinoma cells, the expression of certain genes involved in apoptosis was evaluated: Bax, Bid, Bad, and Bak (pro-apoptotic genes) and Bcl-2 and Bcl-XL (anti-apoptotic genes). Figure 5 displays the effects induced by each sample; one can notice that PBT-5FU produces the most significant up-regulation of the mRNA expression for pro-apoptotic genes, followed by PBT and 5FU.

## 4. Discussion

The gut microbiota as well as the microbiota-derived metabolites have revealed a significant impact on the host immune homeostasis at both the local and the systemic level by causing changes of cell and protein expression which influence systemic inflammation and immune homeostasis [17]. In the colon, direct contact with probiotics has the ability to fight postoperative infectious complications and to reduce the adverse effects of chemotherapy, thus qualifying overall as a potential therapy against the early stages colorectal carcinoma [18]. In addition, probiotics are able to alleviate chronic colon inflammations; *Lactobacillus* sp. proved to be the most effective in improving the symptoms of inflammatory bowel disease [19]. Certain probiotic strains significantly modify the gut microbiota and fight bacterial pathogens such as *Fusobacterium*, which are strongly associated with colorectal cancer proliferation; moreover, probiotics decreased pneumonia as well as the need for postoperative mechanical ventilation [20]. Equally important is their ability to prevent metastasis in colon and other types of cancer, such as ovarian, pancreatic, or breast cancer [6]. Therefore, in the current study, the probiotic anticancer benefits were assessed against two colon cancer cell lines and one lung and one liver cancer cell line, in order to comparatively evaluate their antiproliferative effects not only against colon cancer, but also systemic malignancies.

HT-29, the human colorectal adenocarcinoma cell line, was the first established (1964) colon cancer cell line of human origin used as a model in the study of human colorectal cancers. The cells are known to possess specific characteristics: (i) they express functional receptors for hormones and peptides; (ii) they can synthesize the receptor of dimeric immunoglobulin A; (iii) they can be differentiated in culture under the impact of differentiation inducers (sodium butyrate, dimethyl sulfoxide, etc.); (iv) they possess the capacity to express features of enterocytes and mucus-producing cells and to secrete metabolites, growth factors, pro-angiogenic factors, cytokines, and other factors that sustain cellular survival; and (v) they maintain their cellular properties unchanged even after 100 passages [21]. While the HT29 cell line displays an intermediate ability to differentiate, the other colon cancer cell line, HCT116, is a highly aggressive cell line, almost completely lacking the ability to differentiate [22]. During this study, the data showed a significant cytotoxic activity of PBT against HT29 colon adenocarcinoma cells, in which it combines with the anticancer activity of 5FU in an additive manner. In turn, when the combination of PBT and 5FU was applied to the HCT116 colon cancer cells, the two agents displayed antagonistic effects, with the overall cytotoxic activity being clearly inferior to 5FU alone. In HT29 cells, the results are consistent with those reported in 2016 by An and Ha, who established that *Lactobacillus plantarum* was able to selectively inhibit 5FU-resistent HT29 cells; however, they also reported a similar behavior in HCT116 cells, which contradicts the findings of the current study [23]. Other authors reported no evidence for a clear anticancer activity of probiotics but did report, rather, their intervention against the side effects of chemotherapy drugs while not interfering with their antineoplastic properties [24]. A possible explanation for the higher activity of PBT against HT29 cells is the presence of *C. butyricum* in the PBT combination, which has the ability to modulate mucus production and to induce the glycosylation of mucins in HT29 cells, which contain a glycosylated mucus layer [25]. Therefore, one may assume that the mucus glycan was the targeted site of this bacterium; on the other hand, significant differences were reported between well-differentiated and undifferentiated cell lines in terms of glycan biosynthesis, resulting in the presence of I-branched and sialyl Lewis x/a epitope-bearing glycans in colon-like cell lines such as HT29, versus truncated α2,6-core sialylated glycans in undifferentiated cells such as HCT116 [26]. In addition, within the *Lactobacillus* sp. there is a compositional and structural diversity which significantly influences their antiproliferative activity, such as with the relative proportions of the individual monosaccharides in the produced exopolysaccharides; however, the level of their antiproliferative effect is time-dependent as it was recorded in the current experiment [27]. Another contributing factor to the different cytotoxic activity of PBT against the two colon cancer cell lines is their differences in terms of epigenetic and genetic features; they possess a different status of the KRAS gene with the HT29 cells harboring the wild type of KRAS, while the HCT116 cells contain a mutated type which enables the activation of the KRAS signaling pathway, thus achieving high oncogenic potential and aggressivity. In addition to the KRAS gene, the two colon cancer cell lines also differ in several other cancer critical genes; moreover, the HCT116 cell line contains high proportions of dormant cells (G0/G1 phase), while the HT29 cell line presents more cells in active phases of the cell cycle (S/G2/M), which can be more efficiently attacked by anticancer agents [28]. Collectively, these data may explain the selective cytotoxic activity of PBT against HT29 cells. However, no explanation was found for the antagonistic relationship between PBT and 5FU on HCT116 cells; these findings require further studies.

In A549 lung carcinoma cells, the application of PBT revealed modest anticancer effects; similar antagonistic interactions with 5FU were reported for the PBT-5FU combination. Controversially, using different probiotic strains (*Bifidobacterium* sp.), Ahn et al. reported increased cell death in A549 cells [29]; similarly, *Bacillus polyfermenticus* inhibited in vitro cultured A549 cells alongside other cell lines [30]. However, overall, there are very few studies conducted in the literature on other cancer cell lines than colon; as an example, the antiproliferative activity of three species of *Enterococcus* against several cancer cell lines, including A549, was first described by Sharma et al. in 2018 [31]. The current study could not find evidence for the anticancer effects of the two bacterial strains used against lung cancer cells; the literature reports a retrospective evaluation study on patients with advanced lung cancer which showed significantly prolonged patient survival as a result of supplemental therapy with *C. butyricum*. However, as those outcomes were reported following an in vivo study, one may assume that the immune response was involved in the anticancer activity of the probiotic [32].

Probiotics have been reported to mitigate the risks of hepatocellular carcinoma in vivo [33]; moreover, certain strains of *Streptococcus salivarius* showed the ability to inhibit proliferation in HepG2 cells [34]. In animals, *L. acidophilus* showed antitumor effects against hepatocellular carcinoma; however, in humans, to the best of our knowledge, there are no studies in this regard [35]. However, these effects in vivo can be explained by an enhancement of the antitumor immune response as a result of probiotic administration, thus resulting in tumor inhibition; lacking an immune response, the in vitro environment could not emphasize the anticancer effect of the probiotic [2].

The results of the cell viability tests were confirmed in HT29 cells through microscopic examination at the end of the 24, 48, and 72 h of treatment when the most significant changes, such as the rounding of cells and the decrease in confluence, occurred after 72 h for the PBT-5FU combination. In accordance with Pidgeon et al., morphological changes, such as the rounding of cells and the loss of confluency and adhesion, are the hallmarks of apoptosis [36], which is considered a promising target in cancer treatment [37]. The difference between HT-29 and HCT-116 colorectal carcinoma cells in terms of their individual reaction to 5FU treatment was previously reported by Tawfik et al. as a time-dependent process; the prolonged exposure of colon cancer cells to 5-fluorouracil nanoparticles improves their anticancer activity [38]. Furthermore, another study highlighted that the autophagy process is activated when HT-29 cells are exposed to 5FU for longer periods of time [39]. Moreover, Akhdar et al. found that 5FU activates the Nrf2-ARE signaling pathway in HT-29 cells, a mechanism associated with the chemoresistance of the HT-29 cells to 5FU treatment [40]. With regard to the antitumor activity of probiotics on the HT-29 colorectal carcinoma cell line, numerous studies have demonstrated that probiotic bacteria exhibit both cytotoxic and pro-apoptotic effects [41,42,43], thus validating the results of the current study.

In order to identify the underlying mechanism of cell death, the Hoechst 33342 staining was conducted on HT29 cells; the Hoechst 33342 dye is specifically used to stain the nuclei of eukaryotic living or fixed cells due to its binding to DNA, which results in blue fluorescent stains [44]. Both PBT and 5FU alone induced chromatin condensation, while their combination led to chromatin condensation, nuclear fragmentation, and membrane blebbing, all indicators of apoptotic processes. Apoptosis is the programmed cell death, which evolves with characteristic morphological cell changes, resulting in cell clearance from the body with minimal tissue injuries; it differs from necrosis, which represents the uncontrolled cell death as a result of a damaging process and triggers tissue injuries. The failure of the normal apoptotic process may result in malignant processes; therefore, apoptosis inducers may be used as efficient chemotherapeutics [45]. The results of the current study show an apoptotic activity of the tested probiotic strains as well as their combination with 5FU and are consistent with previously published data. *Lactobacillus rhamnosus* was revealed to produce the p8 protein, which leads to apoptotic cells [46], while *L. brevis* and *L. paracasei* inhibited HT-29 cell proliferation and induced apoptosis in a time-, dose-, and strain-dependent manner [47]. Yue et al. showed very recently that *L. acidophilus* exhibited significant antiproliferative effects in HT29 and Caco-2 cells in a dose- and time-dependent manner; moreover, the apoptotic process induced in HT29 cells increased with time, as reflected by an increased amount of blue fluorescence of the cells [48]. *Lactobacillus* spp. induced selective cytotoxic effects on leukemia and colon tumor cells through pro-apoptotic activities as well as anti-inflammatory effects on macrophages [5]. On the other hand, the butyrate produced by *C. butyricum* was able to induce apoptosis and reduce cell viability in Caco-2 cells in a dose-dependent manner [49]. The pro-apoptotic activity of probiotics might be useful as both a treatment and an adjuvant against cancer.

In order to further assess the antiproliferative activity of probiotics in terms of molecular mechanisms and taking into account the results of the nuclear morphology evaluation which indicated apoptosis induction, the expression of Bax, Bid, Bad, and Bak (pro-apoptotic genes) and Bcl-2 and Bcl-XL (anti-apoptotic genes) was quantified by means of RT-qPCR on HT29 cells, which showed the higher cytotoxicity effects during MTT tests. The outcome of each apoptotic phase is regulated by several genes and their interconnections, with the major contribution of the mitochondria as well as the miRNAs, which act as key factors in the apoptotic process [50]. The central regulators within the intrinsic apoptotic pathway belong to the Bcl-2 protein family, whose members are strongly interconnected; the Bcl-2 family containing pro-apoptotic (Bid, Bad, Bax, and Bak) and anti-apoptotic proteins (Bcl-2, Bcl-XL, Mcl-1, Bcl-w, and A1/Bfl1) often display a deregulation in cancer, and their targeting has triggered the development of new anticancer agents [51]. In the current study, one can clearly see that both the anticancer drug 5FU and the probiotics significantly stimulate the expressions of the Bax, Bid, Bad and Bak proteins, while inhibiting the anti-apoptotic Bcl-2 and Bcl-XL proteins. The most important observation is that the probiotics exhibited a similar ability to act on both types of proteins as the synthetic drug; however, the up-regulation of the pro-apoptotic proteins is more significant compared to the down-regulation of the anti-apoptotic proteins. When analyzing the pro-apoptotic up-regulation of Bax and Bad, the data showed that the combination of PBT and 5FU induces a stronger effect compared to PBT alone, which in turn proved more efficient than the conventional drug; a similar pattern can be noticed for Bad and Bak but with smaller differences in the recorded pro-apoptotic effects. In terms of anti-apoptotic down-regulation, the synthetic drug proved more efficient compared to either its combination with PBT or PBT alone; nonetheless, both PBT and 5FU, or their combination, induced a significantly lower expression of Bcl-XL compared to the control. Collectively, the data revealed that probiotics are able to induce intrinsic apoptosis through the up-regulation of pro-apoptotic proteins and the down-regulation of anti-apoptotic proteins; however, the combination of PBT with 5FU did not produce synergic effects compared to the individual components. Taking into account that PBT exhibited stronger pro-apoptotic effects than the synthetic 5FU drug, the administration of probiotics may produce better clinical outcomes than conventional chemotherapy due to the avoidance of side effects. The reported results are consistent with previously published data on *Lactobacillus acidophilus*, which induced the apoptosis of HT29 cells in a dose- and time-dependent manner through the up-regulation of Bax, Caspase-9, and Caspase-3 and the down-regulation of Bcl-2 [42]. Similarly, another study revealed a cytotoxic activity of *Lactobacillus* spp. on HT29 cells in a time-, dose-, and strain-dependent manner, with the heat-killed probiotic bacteria acting as apoptosis inducers through increased expressions of Bax, caspase-3, and caspase-9 mRNA levels and reduced expressions of Bcl2 [47]; these results were confirmed in an in vivo study on xenografted BALB/c nude mice, where species of *Lactobacillus* were able to inhibit the growth of colorectal cancer [52]. In addition, *C. butyricum* decreased the proliferation and increased the apoptosis of intestinal tumor cells but through the modulation of the Wnt signaling pathway [53]. One can conclude that certain strains of probiotics, such as *Lactobacillus sporogenes* and *Clostridium butyricum*, are able to effectively fight the proliferation of colon cancer cells through intrinsic apoptosis induction, their antitumor potential being comparable to conventional anticancer drugs such as 5FU; simultaneously, the two strains of probiotics were able to add to the pro-apoptotic effect of the chemotherapy drug but could not achieve a synergistic activity.

## 5. Conclusions

Effective cancer treatment is still a goal only glimpsed and not yet achieved due to the ever-evolving nature of the pathology itself, which poses numerous challenges and requires complex research. Probiotics have showed promising anticancer effects which, combined with their ability to fight the side effects of synthetic drugs, may provide potential useful treatments in the future. Two strains of probiotics, *L. sporogenes* and *C. butyricum*, were tested on colon, lung, and liver cancer cells, where cytotoxic effects were noticed in particular on the intermediate differentiated HT29 colon cell line. The studies at the cellular level revealed the occurrence of apoptosis under the effect of the probiotic mix, as indicated by the nuclear morphology assessment by means of Hoechst 33342 staining. In addition, at the molecular level, the expression of the pro-apoptotic markers was significantly increased, while the anti-apoptotic markers displayed a decreasing tendency. Moreover, the probiotic mix revealed a cytotoxic activity comparable to the synthetic drug 5FU, an activity which was also validated at the molecular level by the expression of pro- and anti-apoptotic markers. Collectively, the experimental data show that probiotics have the ability to efficiently fight cancer proliferation; the combination of probiotics with 5FU induced additive cytotoxic effects. Therefore, one can conclude that the two strains of probiotic bacteria may serve as a potential anticancer treatment, particularly against colon cancer. Further studies should reveal their efficiency in vivo and eventually in clinical settings.

## Figures and Tables

**Table 1:** CDI values for the two agents, PBT and 5FU, when used as mixture.

Cell Line	Time of Exposure (h)	CDI Value
HT29	24	0.97
	48	0.92
	72	0.95
HCT116	24	1.23
	48	1.56
	72	1.74
A549	24	1.25
	48	1.85
	72	1.35
HepG2	24	N/A
	48	N/A
	72	N/A

## Data Availability

The data presented in this study are available on request from the corresponding author.

## References

[1] Cancer. https://www.who.int/news-room/fact-sheets/detail/cancer.

[2] Lu K., Dong S., Wu X., Jin R., Chen H. (2021). Probiotics in Cancer. Front. Oncol..

[3] Nataraj B.H., Ali S.A., Behare P.V., Yadav H. (2020). Postbiotics-parabiotics: The new horizons in microbial biotherapy and functional foods. Microb. Cell Factories.

[4] Peng M., Tabashsum Z., Anderson M., Truong A., Houser A.K., Padilla J., Akmel A., Bhatti J., Rahaman S.O., Biswas D. (2020). Effectiveness of probiotics, prebiotics, and prebiotic-like components in common functional foods. Compr. Rev. Food Sci. Food Saf..

[5] Śliżewska K., Markowiak-Kopeć P., Śliżewska W. (2020). The Role of Probiotics in Cancer Prevention. Cancers.

[6] Sankarapandian V., Venmathi Maran B.A., Rajendran R.L., Jogalekar M.P., Gurunagarajan S., Krishnamoorthy R., Gangadaran P., Ahn B.-C. (2022). An Update on the Effectiveness of Probiotics in the Prevention and Treatment of Cancer. Life.

[7] Vodenkova S., Buchler T., Cervena K., Veskrnova V., Vodicka P., Vymetalkova V. (2020). 5-fluorouracil and other fluoropyrimidines in colorectal cancer: Past, present and future. Pharmacol. Ther..

[8] Longley D.B., Harkin D.P., Johnston P.G. (2003). 5-Fluorouracil: Mechanisms of action and clinical strategies. Nat. Rev. Cancer.

[9] Blondy S., David V., Verdier M., Mathonnet M., Perraud A., Christou N. (2020). 5-Fluorouracil resistance mechanisms in colorectal cancer: From classical pathways to promising processes. Cancer Sci..

[10] Baldwin C., Millette M., Oth D., Ruiz M.T., Luquet F.-M., Lacroix M. (2010). Probiotic Lactobacillus Acidophilus and L. Casei Mix Sensitize Colorectal Tumoral Cells to 5-Fluorouracil-Induced Apoptosis. Nutr. Cancer.

[11] Śliżewska K., Chlebicz-Wójcik A. (2020). Growth Kinetics of Probiotic Lactobacillus Strains in the Alternative, Cost-Efficient Semi-Solid Fermentation Medium. Biology.

[12] Zhou M., Yuan W., Yang B., Pei W., Ma J., Feng Q. (2022). *Clostridium butyricum* inhibits the progression of colorectal cancer and alleviates intestinal inflammation via the myeloid differentiation factor 88 (MyD88)-nuclear factor-kappa B (NF-κB) signaling pathway. Ann. Transl. Med..

[13] Farcas C.G., Dehelean C., Pinzaru I.A., Mioc M., Socoliuc V., Moaca E.-A., Avram S., Ghiulai R., Coricovac D., Pavel I. (2020). Thermosensitive Betulinic Acid-Loaded Magnetoliposomes: A Promising Antitumor Potential for Highly Aggressive Human Breast Adenocarcinoma Cells Under Hyperthermic Conditions. Int. J. Nanomed..

[14] Pinzaru I., Chioibas R., Marcovici I., Coricovac D., Susan R., Predut D., Georgescu D., Dehelean C. (2021). Rutin Exerts Cytotoxic and Senescence-Inducing Properties in Human Melanoma Cells. Toxics.

[15] Coricovac D., Dehelean C.A., Pinzaru I., Mioc A., Aburel O.-M., Macasoi I., Draghici G.A., Petean C., Soica C., Boruga M. (2021). Assessment of Betulinic Acid Cytotoxicity and Mitochondrial Metabolism Impairment in a Human Melanoma Cell Line. Int. J. Mol. Sci..

[16] Soica C., Oprean C., Borcan F., Danciu C., Trandafirescu C., Coricovac D., Crăiniceanu Z., Dehelean C., Munteanu M. (2014). The Synergistic Biologic Activity of Oleanolic and Ursolic Acids in Complex with Hydroxypropyl-γ-Cyclodextrin. Molecules.

[17] Liu X., Cheng Y., Zang D., Zhang M., Li X., Liu D., Gao B., Zhou H., Sun J., Han X. (2021). The Role of Gut Microbiota in Lung Cancer: From Carcinogenesis to Immunotherapy. Front. Oncol..

[18] Dikeocha I.J., Al-Kabsi A.M., Eid E.E.M., Hussin S., Alshawsh M.A. (2021). Probiotics supplementation in patients with colorectal cancer: A systematic review of randomized controlled trials. Nutr. Rev..

[19] Ahn S.-I., Cho S., Choi N.-J. (2020). Effect of dietary probiotics on colon length in an inflammatory bowel disease–induced murine model: A meta-analysis. J. Dairy Sci..

[20] Davoodvandi A., Fallahi F., Tamtaji O.R., Tajiknia V., Banikazemi Z., Fathizadeh H., Abbasi-Kolli M., Aschner M., Ghandali M., Sahebkar A. (2021). An Update on the Effects of Probiotics on Gastrointestinal Cancers. Front. Pharmacol..

[21] Martínez-Maqueda D., Miralles B., Recio I. (2015). HT29 Cell Line. the Impact of Food Bioactives on Health.

[22] Yeung T.M., Gandhi S.C., Wilding J.L., Muschel R., Bodmer W.F. (2010). Cancer stem cells from colorectal cancer-derived cell lines. Proc. Natl. Acad. Sci. USA.

[23] An J., Ha E.-M. (2016). Combination Therapy of Lactobacillus plantarum Supernatant and 5-Fluouracil Increases Chemosensitivity in Colorectal Cancer Cells. J. Microbiol. Biotechnol..

[24] Chang C.-W., Liu C.-Y., Lee H.-C., Huang Y.-H., Li L.-H., Chiau J.-S.C., Wang T.-E., Chu C.-H., Shih S.-C., Tsai T.-H. (2018). Lactobacillus casei Variety rhamnosus Probiotic Preventively Attenuates 5-Fluorouracil/Oxaliplatin-Induced Intestinal Injury in a Syngeneic Colorectal Cancer Model. Front. Microbiol..

[25] Lili Q., Xiaohui L., Haiguang M., Jinbo W. (2021). *Clostridium butyricum* Induces the Production and Glycosylation of Mucins in HT-29 Cells. Front. Cell. Infect. Microbiol..

[26] Madunić K., Zhang T., Mayboroda O.A., Holst S., Stavenhagen K., Jin C., Karlsson N.G., Lageveen-Kammeijer G.S.M., Wuhrer M. (2021). Colorectal cancer cell lines show striking diversity of their O-glycome reflecting the cellular differentiation phenotype. Cell. Mol. Life Sci..

[27] Tukenmez U., Aktas B., Aslim B., Yavuz S. (2019). The relationship between the structural characteristics of lactobacilli-EPS and its ability to induce apoptosis in colon cancer cells in vitro. Sci. Rep..

[28] Olejniczak A., Szaryńska M., Kmieć Z. (2018). In vitro characterization of spheres derived from colorectal cancer cell lines. Int. J. Oncol..

[29] An J., Kim H., Yang K.M. (2020). An Aqueous Extract of a Bifidobacterium Species Induces Apoptosis and Inhibits Invasiveness of Non-Small Cell Lung Cancer Cells. J. Microbiol. Biotechnol..

[30] Ma E.L., Choi Y.J., Choi J., Pothoulakis C., Rhee S.H., Im E. (2010). The anticancer effect of probiotic Bacillus polyfermenticus on human colon cancer cells is mediated through ErbB2 and ErbB3 inhibition. Int. J. Cancer.

[31] Sharma P., Kaur S., Kaur R., Kaur M., Kaur S. (2018). Proteinaceous Secretory Metabolites of Probiotic Human Commensal Enterococcus hirae 20c, E. faecium 12a and L12b as Antiproliferative Agents Against Cancer Cell Lines. Front. Microbiol..

[32] Pizzo F., Maroccia Z., Hammarberg Ferri I., Fiorentini C. (2022). Role of the Microbiota in Lung Cancer: Insights on Prevention and Treatment. Int. J. Mol. Sci..

[33] Thilakarathna W.P.D.W., Rupasinghe H.P.V., Ridgway N.D. (2021). Mechanisms by Which Probiotic Bacteria Attenuate the Risk of Hepatocellular Carcinoma. Int. J. Mol. Sci..

[34] Srikham K., Daengprok W., Niamsup P., Thirabunyanon M. (2021). Characterization of Streptococcus salivarius as New Probiotics Derived From Human Breast Milk and Their Potential on Proliferative Inhibition of Liver and Breast Cancer Cells and Antioxidant Activity. Front. Microbiol..

[35] Jeong J.-J., Park H.J., Cha M.G., Park E., Won S.-M., Ganesan R., Gupta H., Gebru Y.A., Sharma S.P., Lee S.B. (2022). The Lactobacillus as a Probiotic: Focusing on Liver Diseases. Microorganisms.

[36] Pidgeon G.P., Kandouz M., Meram A., Honn K. (2002). V Mechanisms controlling cell cycle arrest and induction of apoptosis after 12-lipoxygenase inhibition in prostate cancer cells. Cancer Res..

[37] Pfeffer C., Singh A. (2018). Apoptosis: A Target for Anticancer Therapy. Int. J. Mol. Sci..

[38] Tawfik E., Ahamed M., Almalik A., Alfaqeeh M., Alshamsan A. (2017). Prolonged exposure of colon cancer cells to 5-fluorouracil nanoparticles improves its anticancer activity. Saudi Pharm. J..

[39] Sasaki K., Tsuno N.H., Sunami E., Tsurita G., Kawai K., Okaji Y., Nishikawa T., Shuno Y., Hongo K., Hiyoshi M. (2010). Chloroquine potentiates the anti-cancer effect of 5-fluorouracil on colon cancer cells. BMC Cancer.

[40] Akhdar H., Loyer P., Rauch C., Corlu A., Guillouzo A., Morel F. (2009). Involvement of Nrf2 activation in resistance to 5-fluorouracil in human colon cancer HT-29 cells. Eur. J. Cancer.

[41] Chen Z.-Y., Hsieh Y.-M., Huang C.-C., Tsai C.-C. (2017). Inhibitory Effects of Probiotic Lactobacillus on the Growth of Human Colonic Carcinoma Cell Line HT-29. Molecules.

[42] Guo Y., Zhang T., Gao J., Jiang X., Tao M., Zeng X., Wu Z., Pan D. (2020). Lactobacillus acidophilus CICC 6074 inhibits growth and induces apoptosis in colorectal cancer cells in vitro and in HT-29 cells induced-mouse model. J. Funct. Foods.

[43] Nouri Z., Karami F., Neyazi N., Modarressi M.H., Karimi R., Khorramizadeh M.R., Taheri B., Motevaseli E. (2016). Dual Anti-Metastatic and Anti-Proliferative Activity Assessment of Two Probiotics on HeLa and HT-29 Cell Lines. Cell J..

[44] Almassalha L.M., Bauer G.M., Chandler J.E., Gladstein S., Cherkezyan L., Stypula-Cyrus Y., Weinberg S., Zhang D., Thusgaard Ruhoff P., Roy H.K. (2016). Label-free imaging of the native, living cellular nanoarchitecture using partial-wave spectroscopic microscopy. Proc. Natl. Acad. Sci. USA.

[45] D’Arcy M.S. (2019). Cell death: A review of the major forms of apoptosis, necrosis and autophagy. Cell Biol. Int..

[46] Kim B.-K., Yoon Y.-S., Ryu Y., Chung M.-J. (2021). Probiotic-derived p8 protein induce apoptosis via regulation of RNF152 in colorectal cancer cells. Am. J. Cancer Res..

[47] Karimi Ardestani S., Tafvizi F., Tajabadi Ebrahimi M. (2019). Heat-killed probiotic bacteria induce apoptosis of HT-29 human colon adenocarcinoma cell line via the regulation of Bax/Bcl2 and caspases pathway. Hum. Exp. Toxicol..

[48] Yue Y., Wang S., Shi J., Xie Q., Li N., Guan J., Evivie S.E., Liu F., Li B., Huo G. (2022). Effects of Lactobacillus acidophilus KLDS1.0901 on Proliferation and Apoptosis of Colon Cancer Cells. Front. Microbiol..

[49] Cassir N., Benamar S., La Scola B. (2016). *Clostridium butyricum*: From beneficial to a new emerging pathogen. Clin. Microbiol. Infect..

[50] Cavalcante G.C., Schaan A.P., Cabral G.F., Santana-da-Silva M.N., Pinto P., Vidal A.F., Ribeiro-dos-Santos Â. (2019). A Cell’s Fate: An Overview of the Molecular Biology and Genetics of Apoptosis. Int. J. Mol. Sci..

[51] Leibowitz B., Yu J. (2010). Mitochondrial signaling in cell death via the Bcl-2 family. Cancer Biol. Ther..

[52] Kim S.-J., Kang C.-H., Kim G.-H., Cho H. (2022). Anti-Tumor Effects of Heat-Killed L. reuteri MG5346 and L. casei MG4584 against Human Colorectal Carcinoma through Caspase-9-Dependent Apoptosis in Xenograft Model. Microorganisms.

[53] Chen D., Jin D., Huang S., Wu J., Xu M., Liu T., Dong W., Liu X., Wang S., Zhong W. (2020). *Clostridium butyricum*, a butyrate-producing probiotic, inhibits intestinal tumor development through modulating Wnt signaling and gut microbiota. Cancer Lett..

